# Editorial: The role of education in raising awareness towards antimicrobial resistance (AMR)

**DOI:** 10.3389/fmicb.2024.1444502

**Published:** 2024-06-26

**Authors:** Enrique Castro-Sánchez, Hemda Garelick, Maria Teresa Pérez-Gracia, Rustam Aminov

**Affiliations:** ^1^College of Business, Arts, and Social Sciences, Brunel University London, Uxbridge, United Kingdom; ^2^NIHR HPRU in Healthcare-Associated Infection and Antimicrobial Resistance, Imperial College London, London, United Kingdom; ^3^Research Group on Global Health and Sustainable Human Development, University of the Balearic Islands, Palma de Mallorca, Spain; ^4^Faculty of Science and Technology, Department of Natural Sciences, Middlesex University, London, United Kingdom; ^5^Área de Microbiología, Departamento de Farmacia, Instituto de Ciencias Biomédicas, Facultad de Ciencias de la Salud, Universidad Cardenal Herrera-CEU, CEU Universities, Valencia, Spain; ^6^The School of Medicine, Medical Sciences, and Nutrition, University of Aberdeen, Aberdeen, United Kingdom

**Keywords:** antimicrobial resistance (AMR), antimicrobial stewardship (AMS), education, awareness, public health

Antimicrobial resistance (AMR) poses a significant threat to global health, requiring multifaceted strategies to mitigate its spread. Education plays a pivotal role in raising awareness and providing various stakeholders with the knowledge and skills to address various AMR issues. This editorial summarizes the contributions published in the Research Topic “*The role of education in raising awareness towards antimicrobial resistance*” featuring innovative educational strategies and their impact on AMR awareness and stewardship.

The increasing importance of education to underpin and strengthen AMR interventions cannot be overstated. Effective educational strategies not only enhance the performance of prescribers and raise awareness among citizens, but are also vital to foster a culture of stewardship and responsible antimicrobial use. These strategies remain crucial given the gaps highlighted by publications in this Research Topic. For example, in the systematic review by Atalay and Gelaw, the authors synthesized data on the knowledge, attitudes, and practices regarding AMR across different populations in Africa, including the public, patients, healthcare workers, pet owners, and students. The analysis of 39 studies from various African countries identified suboptimal levels of AMR knowledge and related practices, highlighting the need for targeted educational interventions and collaborative efforts.

In this regard, several authors in this Research Topic reported on educational interventions. For example, as described by Njeru et al., the collaborative efforts between the USAID's Infectious Disease Detection and Surveillance project and the National Antimicrobial Stewardship Interagency Committee of Kenya allowed the development of a successful AMR surveillance curriculum, which was intended for in-service healthcare workers from various sectors, including human and animal health, thus implementing the “One Health” approach. Initial results from training sessions showed substantial improvements in participants' knowledge and skills, as evidenced by increased pre- and post-test scores. These targeted training programs also significantly improved healthcare workers' understanding of AMR and their capacity to implement effective stewardship practices.

Another way of developing educational models for AMR awareness programmes was presented in this topic by Tyrrell, Ayanikkad et al.. They described a project, which involved the postgraduate research training, primary school science education, outreach activities and public engagement components. Masters students were tasked with the development of educational activities for primary school pupils, and they produced highly-rated materials such as an interactive card game, with additional benefits for the students such as education, service learning, and public engagement. The project highlighted the powerful impact of this combinatorial approach toward AMR education. The implementation of participatory approaches in AMR education was the focus of another study by the same group (Tyrrell, Hatch et al.). They used co-production workshops with primary stakeholders, including university researchers, engagement professionals, school teachers, and web designers, to develop tailored educational content on the Web. The success of this initiative highlights the value of involving end-users in the development process to enhance the impact of educational interventions.

Emerging technologies offer new opportunities to enhance the educational efforts. For example, digital platforms and mobile applications can provide accessible and interactive learning experiences, while the analysis of user engagement can help tailor educational content to specific audiences and track the impact of interventions in real-time. The article by the SWICEU team in Spain explored the effectiveness of gamification in educating young people about AMR (Tarín-Pelló et al.). Over 5 years, the team has developed various educational tools based on games such as card games, escape rooms, and digital challenges. These tools were designed to make complex concepts about bacterial resistance and antibiotic use accessible and engaging. The project demonstrated significant success in raising awareness and encouraging scientific vocations among pre-university and university students through interactive and fun learning methods.

Despite the advancements in educational approaches, several gaps remain. One significant challenge is the evaluation of the impact of educational interventions. Many studies rely on immediate pre- and post-training assessments, which may not capture the long-term effects of education on behavior and practice. There is a need for more longitudinal studies to assess the sustained impact of AMR education, and broader considerations of what meaningful impact should be, including the views of educators, learners, and the wider society.

Additionally, the cost-effectiveness of educational programs is often not thoroughly evaluated. Understanding the economic implications of different educational strategies is crucial for the scaling up of successful interventions and ensuring that resources are efficiently allocated. Future research should focus on developing robust cost-effective analyses to inform policy decisions and optimize the allocation of funding for AMR education initiatives.

In conclusion, the articles in this Research Topic underscore the vital role of education in addressing the global challenge of AMR. Innovative strategies and emerging technologies hold promise for enhancing the effectiveness of educational interventions. However, the long-term impact and cost-effectiveness of these programs must be constantly evaluated to ensure their sustainability and scalability. By addressing these gaps, we can strengthen existing collective efforts to combat AMR and safeguard public health for future generations.

## Author contributions

EC-S: Writing – original draft. HG: Writing – review & editing. MP-G: Writing – review & editing. RA: Writing – review & editing.

